# Alpha-like subunits of multisubunit RNA polymerases: insights into structure, function, and disease

**DOI:** 10.1080/15476286.2026.2700806

**Published:** 2026-07-08

**Authors:** Onyinyechi C. Onuoha, Alana E. Belkevich, Ryan J. Palumbo, Bruce A. Knutson

**Affiliations:** a Department of Biochemistry and Molecular Biology, SUNY Upstate Medical University, Syracuse, NY, USA; b Department of Biology, Saint Lawrence University, Canton, NY, USA

**Keywords:** Alpha subunit, RNA polymerase, disease, Transcription, complex assembly

## Abstract

Multisubunit RNA polymerases share a deeply conserved architectural core, yet their regulatory complexity has expanded dramatically over evolution. At the heart of this architecture lies an ancient assembly module formed by the alpha and alpha-like subunits. Originally emerging as a homodimeric scaffold in bacteria, this module was duplicated and partitioned into asymmetric heterodimers in archaea and eukaryotes. Far from serving as inert structural elements, alpha-like subunits function as organizational hubs that coordinate polymerase biogenesis, interface with initiation and regulatory factors, and—in some contexts—operate outside the holoenzyme to influence RNA processing and growth signalling pathways. Recent structural and genetic studies reveal that although the α-motif–mediated dimerization framework is highly conserved, species- and polymerase-specific tuning of alpha-like subunit interaction surfaces has generated functional diversification, with important consequences for assembly fidelity, transcriptional regulation, and disease susceptibility. Mutations in alpha-like subunits shared between RNAPI and RNAPIII underlie a ribosomopathy and leukodystrophy, while altered dosage contributes to oncogenic growth programmes and proliferative signalling. These findings highlight alpha-like subunits as evolutionarily conserved yet adaptable platforms that couple transcriptional capacity to developmental and metabolic demands. By integrating structural, evolutionary, mechanistic, and clinical evidence, this review described the alpha-like subunits as central regulators of polymerase function and as emerging determinants of transcription-linked disease.

## Introduction

Multisubunit RNA polymerases are among the most conserved and essential molecular machines in biology, responsible for the transcription of genes that sustain cellular growth, metabolism, and differentiation. In eukaryotes, three nuclear RNA polymerases (RNAPI, RNAPII, and RNAPIII) carry out specialized transcriptional programmes, whereas bacteria and archaea utilize a single multisubunit enzyme [1–4]. While decades of structural and biochemical work have illuminated the catalytic mechanisms of RNA synthesis, it has become increasingly clear that proper transcription depends not only on enzymatic activity but also on precise polymerase assembly, stable subunit architecture, and regulated interactions with transcription factors and chromatin. Disruption of structural and regulatory features can compromise transcriptional homoeostasis and contribute directly to human disease.

Across all domains of life, a critical architectural feature of multisubunit RNA polymerases is the presence of alpha or alpha-like subunits that form an early assembly scaffold [3]. In bacteria, two identical alpha subunits homodimerize to nucleate polymerase biogenesis, recruit catalytic subunits, and provide flexible interfaces for promoter recognition and transcriptional regulation. In archaea and eukaryotes, this scaffold is composed of heterodimeric alpha-like subunits that retain core dimerization motifs while acquiring polymerase-specific regulatory functions [3,5,6].

Rather than serving merely as passive structural components, alpha-like subunits play important roles in polymerase assembly, transcriptional regulation, and cellular signalling. Their conserved position within RNA polymerases enables coordination of catalytic subunits and integration of regulatory inputs. In some cases, alpha-like subunits perform polymerase-independent functions. Notably, mutations or altered expression of alpha-like subunits are increasingly linked to developmental disorders and cancer, underscoring their biological and clinical relevance [7–9]. Despite their structural conservation and central placement within RNA polymerase complexes, alpha-like subunits have historically received less attention than catalytic subunits or transcription factors, and their broader roles in polymerase regulation and disease remain underappreciated.

In this review, we examine alpha and alpha-like subunits in terms of their structure, evolutionary origin, and functional diversification. We first describe the conserved architectural features that define these subunits across domains of life, then discuss how this scaffold contributes to polymerase assembly and transcriptional control. Finally, we explore how evolutionary plasticity and ‘extrapolymerase’ roles expand their functional roles beyond transcription.

## Structure and evolution of alpha and alpha-like subunits

**Figure 1.**
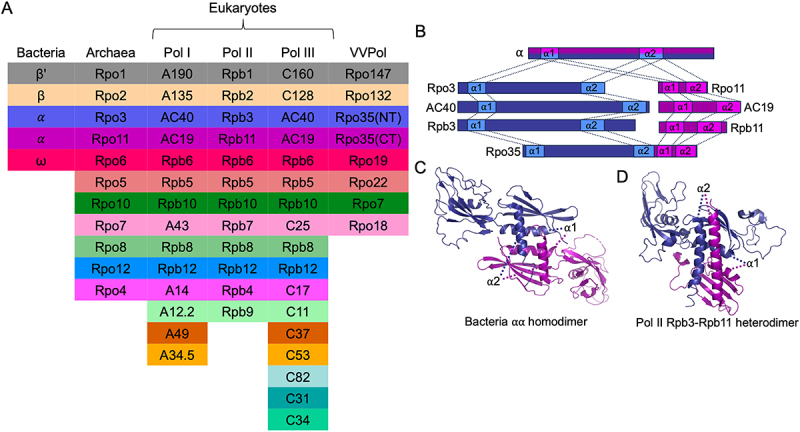
Conservation of RNA polymerase subunits and α-like assembly architecture across domains of life.

**Figure 2.**
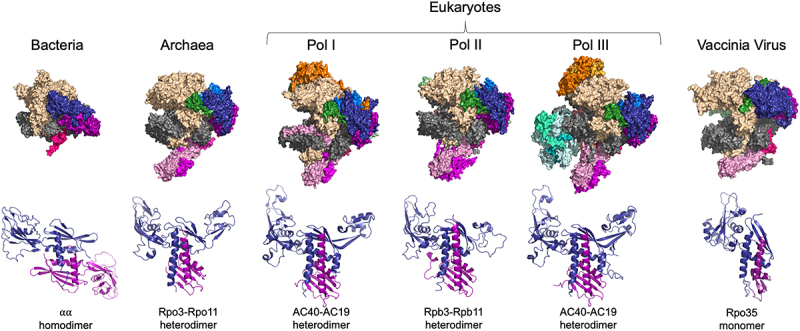
Structural conservation of alpha-like subunits across domains of life.

Alpha and alpha-like subunits form one of the most deeply conserved architectural modules in multisubunit RNA polymerases. Across all domains of life, these factors function as early assembly scaffolds that organize polymerase biogenesis and position regulatory interfaces without directly participating in catalysis [3,10]. In bacteria, this role is fulfilled by a homodimer of identical alpha subunits, whereas in archaea and eukaryotes the same architectural framework is preserved through heterodimeric alpha-like subunits (Figure 1(A)). The bacterial alpha homodimer represents the ancestral assembly module, which was duplicated and partitioned into asymmetric alpha-like heterodimers in archaea and eukaryotes (Figure 1(B)). A combination of subfunctionalization and neofunctionalization likely facilitated this transition, preserving the core alpha-motif mediated dimerization interface while allowing polymerase-specific interfaces and regulatory capacities to emerge alongside increasing transcriptional complexity. This combination of architectural constraint and modular diversification underlies both the essential role of alpha-like subunits in RNA polymerase biogenesis and their recurrent involvement in polymerase assembly defects, transcriptional dysregulation, and human disease. Despite evolutionary divergence, alpha-like subunits are consistently positioned at the periphery of the polymerase core, at the junction of the two largest catalytic subunits, where they serve as a conserved architectural bridge linking the catalytic centre to the broader enzyme scaffold (Figure 2).

(A) Comparative overview of RNA polymerase subunits from bacteria, archaea, eukaryotic RNA polymerases I–III, and Vaccinia virus RNA polymerase (VVPol). Orthologous subunits are aligned by evolutionary and structural correspondence. Each subunit family is assigned a distinct colour to illustrate conserved modules across polymerases and highlight lineage-specific expansions in eukaryotic enzymes. (B) Domain organization of the α-motif–containing subunits across archaeal and eukaryotic polymerases. The bacterial α subunit contains two α-motif domains (α1 and α2) that are evolutionarily partitioned into separate proteins in archaeal and eukaryotic polymerases (e.g. Rpo3/Rpo11 in archaea and Rpb3/Rpb11 or AC40/AC19 in eukaryotes). Finally vaccinia virus Rpo35 is a hybrid containing four α-motif in a single protein. Dotted lines indicate evolutionary relationships between these conserved α-motif domains. (**C-D**) Ribbon representation of the (C) bacterial αα homodimer and (D) eukaryotic Pol II Rpb3-Rpb11 heterodimer. Dotted lines indicate the evolutionarily conserved α-motifs.

Surface representations of representative RNA polymerases from bacteria, archaea, eukaryotes, and *vaccinia virus* reveal conserved placement of alpha-like subunits within the enzyme core. Ribbon representations of the corresponding alpha-like dimers are shown below, with large alpha-like subunits in dark blue and smaller partners in purple. Despite evolutionary divergence, the alpha-like complex occupies a comparable architectural position that bridges catalytic subunits and supports core assembly.

## Bacterial alpha subunits: a homodimeric assembly scaffold

**Figure 3.**
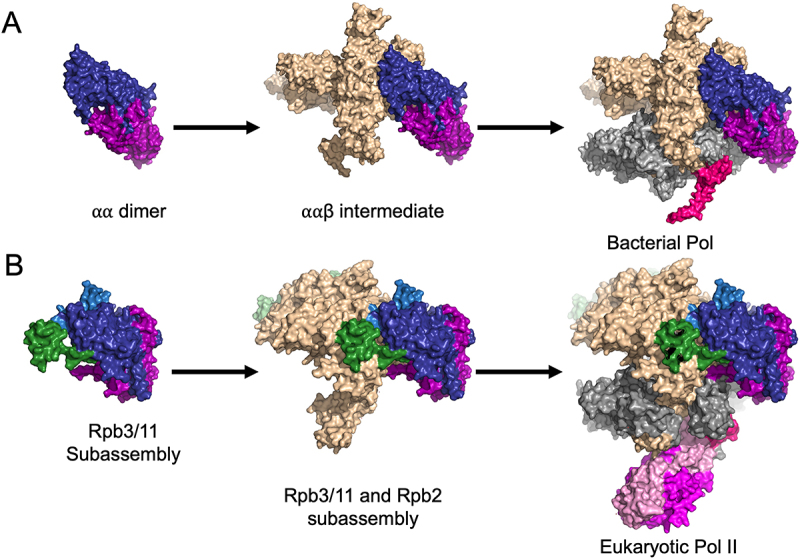
Comparative assembly pathways of bacterial and eukaryotic RNA polymerases.

Prokaryotic RNA polymerase contains two identical alpha subunits that homodimerize to form the earliest stable assembly intermediate of the enzyme [11] (Figure 3(A)). In *E. coli*, the alpha subunit consists of two independently folded domains: an N-terminal domain (αNTD) and a C-terminal domain (αCTD) connected by a short flexible, intrinsically disordered linker [12]. The αNTD mediates homodimerization and interactions with the β and β′ polymerase subunits, while the αCTD engages promoter DNA and transcription factors [13–17].

(A) Structural model of the bacterial RNA polymerase assembly pathway. (B) Proposed assembly pathway for eukaryotic RNA polymerase II. In contrast to the bacterial αα homodimer scaffold, the α-like modules form a heteromeric Rpb3/Rpb11 subassembly that also contains Rpb10 and Rpb12, which associates with Rpb2 and additional subunits to generate a larger intermediate that ultimately assembles into the mature RNAPII enzyme. Colours correspond to the conserved subunit families indicated in Figure 1(A).

The αNTD itself is subdivided into two structural regions: domain 1 and domain 2. Homodimerization is driven exclusively by domain 1 through interlocking helices H1 and H3 from each alpha subunit, forming a hydrophobic core known as the α-motif. Structural and biochemical studies have shown that formation of this α2 homodimer represents the earliest stable assembly intermediate in bacterial RNA polymerase biogenesis [11,18,19]. The α-motif generates a symmetric dimerization platform that positions distinct interaction surfaces on opposite sides of the homodimer, thereby creating a scaffold for recruitment of the catalytic β and β′ subunits. Following dimerization, the two alpha subunits adopt functionally asymmetric roles, with one preferentially engaging β and the other β′ [16]. Thus, homodimerization not only stabilizes the alpha subunits but also spatially organizes the interaction surfaces required for stepwise assembly of the RNA polymerase core enzyme. The flexible linker connecting the αNTD and αCTD allows the αCTD to sample multiple spatial positions relative to the core enzyme, providing a structural basis for promoter-specific DNA engagement and dynamic activator-dependent regulation [12]. While the αCTD confers much of the regulatory versatility of bacterial RNA polymerase, the αNTD-defined dimerization and assembly mechanism represents the ancestral function of the alpha subunits that is retained across evolution.

## Archaeal alpha-like subunits

Archaeal RNA polymerases encode two distinct alpha-like subunits, RpoD and RpoL, which form a stable heterodimer that functions similarly to the bacterial alpha homodimer as an early assembly scaffold. This heterodimer preserves the core α-motif–based dimerization architecture while introducing asymmetry and additional stabilization features [20,21]. The larger RpoD subunit contains a conserved dimerization domain and a fold in domain 2 that is structurally homologous to bacterial and eukaryotic alpha-like subunits (Figures 1(B), 2). Notably, archaeal domain 2 includes cysteine residues that form disulphide bonds absent in bacteria, suggesting enhanced stabilization mechanisms tailored to archaeal cellular environments [22].

In many archaeal species, the RpoD subunit also contains a third, ferredoxin-like domain that coordinates one or two iron–sulphur [4Fe–4S] clusters through conserved cysteine ligands. Structural studies place this Fe–S module within the RpoD/RpoL assembly subcomplex but distant from the catalytic centre, consistent with a structural or allosteric role rather than a direct catalytic function [22,23]. Functional analyses support this view. In *Sulfolobus solfataricus*, disruption of Fe–S cluster coordination destabilizes the RpoD subunit and weakens RpoD/RpoL heterodimer formation [22]. In the anaerobe *Methanosarcina acetivorans*, biochemical reconstitution experiments showed that the RpoD subunit binds two oxygen-labile Fe–S clusters; deletion of the Fe–S domain does not prevent initial heterodimer formation but leads to complex instability and aggregation. Moreover, loss of individual clusters impairs productive association of the RpoD/RpoL scaffold with downstream catalytic subunits during later assembly stages [24,25].

Together, these findings support a model in which archaeal alpha-like subunits retain the ancestral bacterial assembly framework while incorporating additional stabilization features through Fe-S cluster coordination. These clusters play important roles in heterodimer stability and productive RNA polymerase assembly. Although the oxygen sensitivity of the Fe-S clusters raises the possibility that polymerase assembly or stability could respond to environmental conditions, direct evidence for a regulatory role remains limited.

## Eukaryotic alpha-like subunits

Eukaryotic RNA polymerases encode two distinct alpha-like heterodimers: one specific to RNAPII and one shared between RNAPI and RNAPIII. In yeast, these are Rpb3/Rpb11 for RNAPII and AC40/AC19 for RNAPI and RNAPIII, with corresponding human orthologs POLR2C/POLR2J and POLR1C/POLR1D, respectively (Figures 1(A), 2). All eukaryotic alpha-like subunits are homologous to the bacterial αNTD and retain the conserved α-motif required for heterodimerization [26–28] (Figure 1(B)). In larger alpha-like subunits, the two α-motifs are separated by approximately 140–200 residues, whereas in smaller subunits the motifs are adjacent, together forming a leucine-zipper–like dimerization interface. High-resolution structures position the alpha-like heterodimer as an architectural hub that bridges core catalytic subunits and shapes polymerase architecture near the clamp and active-centre regions [6,29].

Despite this conserved role, significant divergence exists between RNAPII and RNAPI/III alpha-like subunits. Compared to Rpb11, Rpb3 in RNAPII is more like the bacterial αNTD and contains a zinc-binding loop that is absent in AC40, the shared RNAPI/III paralog [6,28]. Conversely, the AC40 in RNAPI/III contains a third domain that structurally resembles the archaeal ferredoxin-like fold but lacks the cysteine ligands required for Fe–S coordination. This domain likely functions as a structural pseudo-Fe–S module, reinforcing local packing or providing protein-interaction surfaces rather than serving as a metal-binding cofactor. AC40 also possesses an intrinsically disordered N-terminal extension preceding the dimerization domain, which may contribute to flexibility or polymerase-specific regulatory interactions [30].

## Viral multisubunit RNA polymerase alpha-like factor analogs

Notably, analogous alpha-like factor subcomplexes are also encoded by several large cytoplasmic DNA viruses. A particularly striking example of conserved RNA polymerase assembly architecture outside of cellular systems comes from poxviruses, which replicate and transcribe their genomes entirely in the cytoplasm and therefore encode a complete, multisubunit viral DNA-dependent RNA polymerase (vRNAP) [31]. Cryo-EM structures of the *Vaccinia* virus core and complete vRNAP complexes revealed that the viral enzyme closely resembles eukaryotic RNAPII in overall architecture, including conservation of the catalytic cleft, bridge helix, and nucleic-acid binding elements [32,33]. However, *Vaccinia* vRNAP lacks many canonical RNAPII peripheral subunits and instead incorporates virus-specific regulatory factors directly into the polymerase complex. Importantly, the structural platform of the enzyme is formed by a subcomplex comprising Rpo35 and Rpo7, where Rpo35 combines features of both Rpb3 and Rpb11 within a single polypeptide (Figures 1(B), 2), and Rpo7 occupies a position analogous to Rpb10 in RNAPII. Together, these subunits create a stable assembly module that anchors the two largest catalytic subunits and organizes the overall enzyme architecture, thereby performing analogous functions to the Rpb3/11 platform in RNAPII and alpha-like assembly modules in other multisubunit RNA polymerases [32].

Subsequent genomic and structural studies indicate that poxvirus is not unique in this regard but rather represents a broader class of large DNA viruses that encode eukaryotic-like multisubunit DNA-dependent RNA polymerases. African swine fever virus (ASFV) and multiple members of the nucleocytoplasmic large DNA viruses (NCLDVs; including mimiviruses and related giant viruses) encode polymerase subunits homologous to eukaryotic Rpb1/2 as well as smaller structural subunits corresponding to Rpb3/11-like and Rpb5/6-like components [33,34]. Recent structural analyses of ASFV RNA polymerase confirm a conserved multisubunit core organization consistent with this evolutionary relationship, despite major divergence in regulatory interfaces and accessory factors [35,36]. Together, these viral transcription systems demonstrate that a dedicated platform module equivalent to the alpha-like assembly complex is a fundamental organizational requirement of viral multisubunit RNA polymerases. Interestingly, this feature is maintained even when transcription is fully removed from the nucleus, uncoupled from host transcription machinery, and packaged into virions. Thus, viral RNA polymerases illustrate that alpha subunits and biogenesis are evolutionarily constrained.

## Functional roles in transcription and beyond

**Figure 4.**
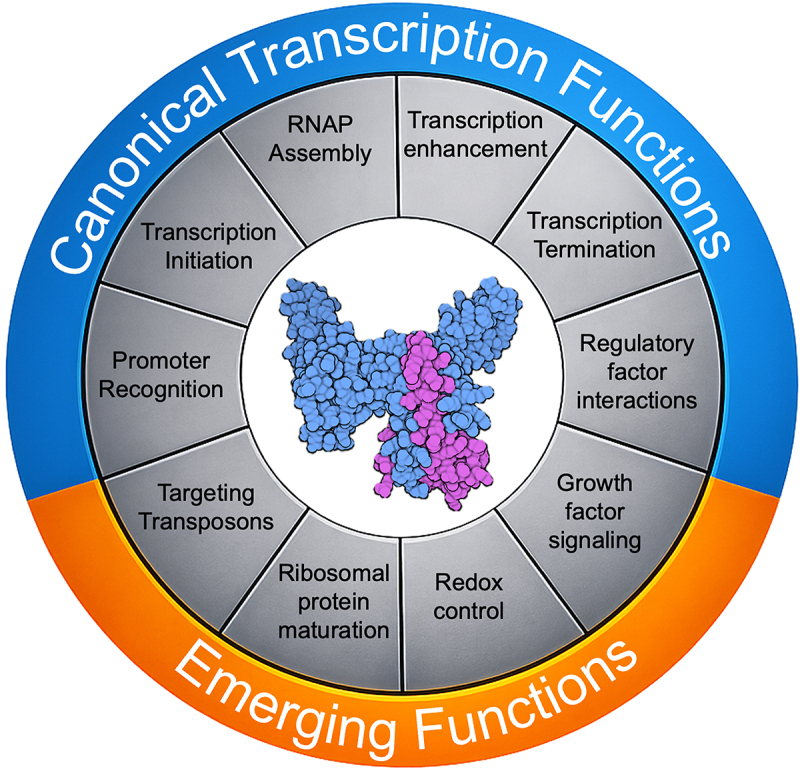
Canonical and emerging functions of alpha-like subunits beyond transcription.

Although alpha and alpha-like subunits are best known for their conserved structural roles within RNA polymerase complexes, genetic, biochemical, and structural studies indicate that they also participate directly in multiple stages of the transcription cycle [37–41]. Their positioning within the RNA polymerase core allows them to influence not only enzyme assembly but also promoter engagement, regulatory factor interactions, and later transitions during elongation and termination. Thus, alpha-like subunits serve as multifunctional platforms that integrate polymerase biogenesis with dynamic regulatory inputs, enabling transcriptional responses to developmental and environmental cues (Figure 4).

Alpha-like subunits perform conserved roles in RNA polymerase assembly and transcription across all domains of life. Canonical functions include homodimerization or heterodimerization, assembly of the RNA polymerase core enzyme, recruitment of catalytic subunits, promoter engagement, interactions with transcriptional regulators, and stabilization of the transcription machinery. These functions are evolutionarily conserved and are essential for RNA polymerase biogenesis and activity. Recent studies have also identified emerging functions of alpha-like subunits that extend beyond their established roles in RNA polymerase assembly and transcription. These include contributions to RNA processing, transposon targeting, growth-associated signalling pathways, and modulation of proliferative programmes. While some of these activities are supported by direct experimental evidence, others represent emerging observations whose underlying molecular mechanisms remain incompletely understood. Together, these findings suggest that alpha-like subunits may influence cellular physiology through both canonical transcription-associated functions and additional regulatory activities that remain an active area of investigation.

## RNA polymerase assembly functions

Across domains, alpha and alpha-like subunits act as a scaffold in RNA polymerase biogenesis. In bacteria, assembly begins with α homodimerization, followed by recruitment of β and then β′ (associated with ω), producing the core enzyme [42] (Figure 3(A)). A recently described Bacillus subtilis assembly intermediate containing α_2_β′ suggests an alternative assembly pathway in which the β subunit is incorporated during the final stages of core enzyme formation. This intermediate has not been observed in *E. coli*, where assembly is thought to proceed through a different sequence of subunit addition. Despite these differences, the α homodimer remains the central assembly scaffold that nucleates formation of the RNA polymerase core and provides the structural foundation upon which the catalytic centre is assembled [43]. In archaea and eukaryotes, polymerase assembly similarly begins with heterodimerization of alpha-like subunits, which then associate with peripheral subunits (eukaryotic Rpb10 and Rpb12; archaeal RpoN and RpoP) to form an early assembly platform that precedes incorporation of the largest catalytic subunits [20,44,45] (Figure 3(B)). The alpha-like heterodimers form at an early and potentially rate-limiting step in polymerase biogenesis, and mutations that destabilize these interfaces impair polymerase assembly upstream of transcriptional defects [46–48].

In eukaryotes, RNA polymerase biogenesis requires dedicated assembly and transport factors, and increasing evidence indicates that alpha-like heterodimers participate directly in these pathways [10,49]. RNA polymerase dissociation and structural studies in yeast identified stable subassemblies containing the second-largest catalytic subunit together with the AC40/AC19 heterodimer and small core subunits, suggesting that alpha-like subunits form early assembly intermediates [50]. Supporting this model, genetic and biochemical analysis of yeast RNAPIII revealed that mutations in the second-largest subunit (C128) that disrupts its interface with AC40/AC19 impaired polymerase assembly and reduced levels of mature RNAPIII complexes. This phenotype was partially rescued by overexpression of the shuttling factor Rbs1, which binds RNAPIII and facilitates nuclear import of assembly intermediates [51]. These findings indicate that eukaryotic RNAPI and III alpha-like heterodimers serve as organizational hubs within cytoplasmic assembly and transport pathways, coordinating productive incorporation of catalytic subunits prior to nuclear targeting. Similar principles apply to RNAPII, where assembly intermediates associate with chaperone and GTPase systems that monitor completion of core subunit incorporation before nuclear import [52,53]. The discovery of distinct subassemblies of alpha subunits and associated factors in eukaryotes further supports a conserved role for alpha-like subunits in coupling polymerase assembly with trafficking and quality control.

## Promoter engagement and regulation

In bacteria, while the −35 element is recognized by σ factors, the αCTD enhances promoter recognition by binding AT-rich UP elements located upstream of this region at a subset of promoters [54]. At a subset of bacterial promoters, transcription initiation is stimulated by activator proteins such as cyclic AMP receptor protein (CRP/CAP), which enhance recruitment and activity of RNA polymerase [55–57]. The αCTD plays a central role in these activation mechanisms by serving as a flexible interaction platform for both promoter DNA and transcriptional activators. Bacterial activator-dependent promoters are commonly classified into three groups based on the position of the activator-binding site and the mechanism of activation.

The distinction between Class I and Class II promoters lies in the nature of their interactions with RNA polymerase. Class I activation is mediated primarily through contacts between activator region 1 (AR1) and the αCTD, whereas Class II promoters additionally involve interactions between activator region 2 (AR2) and the αNTD [58–60]. At Class I promoters, the activator-binding site is located upstream of the −35 element. Activation primarily occurs through protein-protein interactions between the activator and the αCTD, which stabilize RNA polymerase binding and promote transcription initiation [61,62]. At Class II promoters, the activator-binding site overlaps the RNA polymerase binding region. In addition to αCTD-mediated contacts, interactions involving the αNTD contribute to activation by stabilizing promoter complexes and facilitating open-complex formation [63–65]. Class III promoters contain multiple activator-binding sites and are regulated through cooperative interactions between two or more activators. These promoters often employ combinations of Class I and Class II activation mechanisms to achieve synergistic transcriptional activation [59,66].

Activator binding sites vary widely in position and orientation, therefore the dynamic repositioning of the αCTD is essential for effective communication between regulatory proteins and the polymerase. The flexible linker between the αNTD and αCTD allows the αCTD to reposition relative to the core enzyme and engage both DNA and regulatory proteins at variable distances across diverse promoter architectures [62,67,68].

## Polymerase-specific regulatory interactions in eukaryotes

In eukaryotes, alpha-like subunits retain their central assembly role while also mediating polymerase-specific regulatory interactions. In RNAPI, the AC40/AC19 heterodimer interacts tightly with the initiation factor Rrn3 during transcription initiation, positioning this heterodimer at the interface between the core enzyme and initiation machinery [41,69,70]. Because the AC40/AC19 heterodimer lies at the periphery, it is accessible to regulatory contacts, and mutations at this interface could impair initiation without fully disrupting polymerase assembly.

In RNAPII, Rpb3 and Rpb11 interact with the Mediator complex to facilitate promoter engagement and transcription initiation [39,40,71,72]. In addition to these well-established roles within the core transcription machinery, Rpb3 and Rpb11 have been reported to interact with specific transcription factors, suggesting that alpha-like subunits may contribute to specialized transcriptional programmes in certain cellular contexts [73–75]. Although the mechanistic significance of these interactions remains incompletely understood, they point to regulatory functions that extend beyond the structural role of Rpb3 within RNAPII. Notably, the regions of Rpb3 implicated in these interactions are not conserved in the corresponding RNAPI and RNAPIII alpha-like subunits [74], consistent with functional diversification following the evolutionary specialization of the three eukaryotic RNA polymerases.

Alpha and alpha-like subunits also contribute to later stages of transcription. In bacteria, αCTD interacts with the elongation factor NusA, which influences polymerase pausing and participates in termination pathways [37]. In eukaryotes, mutations in Rpb3 and Rpb11 can induce transcriptional readthrough at termination sites. These mutations cluster near the heterodimer interface and do not necessarily disrupt polymerase assembly, suggesting that alpha-like subunits may transmit conformational or regulatory signals relevant to elongation-termination transitions [38]. Overall, the alpha and alpha-like subunits contribute to transcription at multiple levels, ranging from early assembly events to promoter recognition, regulatory factor coupling, and termination control.

## Emerging functions of alpha-like subunits beyond transcription

Recent work in mammalian systems suggests that some alpha-like subunits may participate in biological processes beyond their canonical roles in RNA polymerase assembly and transcription. Although these activities are less well characterized than their established functions within the transcription machinery, emerging evidence indicates that individual alpha-like subunits can influence RNA processing, growth-associated signalling pathways, and other cellular processes under specific conditions (Figure 4). Support for this concept comes from acute degradation studies coupled with multi-omics profiling, which have revealed that depletion of individual RNAPII subunits does not result in uniform transcriptional collapse. Instead, loss of specific subunits produces distinct gene-selective effects on transcription and RNA processing. Notably, depletion of Rpb3, Rpb9, Rpb10, or Rpb11 altered discrete subsets of transcripts, suggesting that individual polymerase subunits can differentially influence transcriptional regulation and RNA maturation [76]. These findings raise the possibility that specific subunits contribute specialized regulatory functions beyond their shared roles in maintaining RNA polymerase integrity and activity.

Extending this concept further, subsequent work identified pools of dissociated RPB3 (dRPB3) in multiple cell types and demonstrated that this free subunit plays a direct role in post-transcriptional regulation. dRPB3 preferentially regulates 3′-end processing of ribosomal protein mRNAs through interactions mediated by its N-terminal domain, engaging the cap-binding complex and recruiting PCF11 via the little elongation complex (LEC) pathway. Importantly, selective degradation of dRPB3 impaired ribosomal protein mRNA maturation without disrupting the integrity of the RNAPII holoenzyme, providing evidence that RPB3 can exert regulatory functions independently of its structural role within the polymerase [77].

Emerging evidence further suggests that alpha-like subunits may participate directly in cytoplasmic growth control pathways, extending their extrapolymerase functions beyond the nucleus. POLR1D has been detected in the cytoplasm, where it associates with mTORC1 signalling complexes at the lysosome and contributes to activation of growth-promoting pathways in cancer cells, including PI3K-Akt and p38 MAPK signalling [78,79]. In cancer cells, POLR1D promotes accumulation of β-catenin and cyclin D1, facilitating G1-S cell-cycle progression [80]. Together, these findings suggest that POLR1D may participate in growth-associated signalling pathways in addition to its established role in RNA polymerase assembly. However, the molecular mechanisms underlying these observations remain incompletely defined.

## Alpha-like factors and transposable elements

Alpha-like subunits are not dedicated regulators of transposable element integration but instead are exploited by mobile elements for targeting. In yeast, the Ty1 integrase (IN1) interacts with AC19 and AC40 to mediate genomic integration of the LTR retrotransposon Ty1, which preferentially inserts within ~1 kb upstream of RNAPIII-transcribed genes [81]. AC40 engages IN1 through a hairpin loop encompassing residues ~108–130 and its C-terminal helix, with AC19 contributing minor stabilizing contacts. Because these subunits are shared between RNAPI and RNAPIII, they provide a conserved docking surface for IN1. Rather than directing integration, however, IN1 co-opts these interactions to access transcription complexes, with RNAPIII-associated assemblies preferentially supporting insertion at RNAPIII loci [82,83]. These findings highlight how Ty1 exploits conserved transcription machinery through the alpha subunits to achieve locus-specific integration.

## Evolutionary plasticity of alpha-like subunit interfaces

A recent study showed that although alpha-like subunits of eukaryotic RNA polymerases are structurally conserved, they employ species- and polymerase-specific interaction mechanisms to form heterodimers, with important consequences for polymerase assembly and disease modelling [84]. Comparing yeast and human RNAPII heterodimers (Rpb3/Rpb11 vs. POLR2C/POLR2J2) and RNAPI/III heterodimers (AC40/AC19 vs. POLR1C/POLR1D) pairs revealed that conserved features such as the alpha-motif and C-terminal helix contribute differently to heterodimerization depending on both polymerase and species [48,84]. Yeast AC19 tolerates mutations with minimal effects on binding or growth, whereas human POLR1D is highly sensitive, with analogous mutations abolishing interaction with POLR1C. Fine mapping further revealed that paralogous subunits rely on distinct residues to heterodimerize within otherwise conserved interfaces. Notably, a humanized yeast AC19 chimera becomes sensitive to clinically relevant mutations, including the TCS-associated human G52E substitution (G73E in yeast), which disrupts AC40 binding and compromises RNAPI and RNAPIII complex integrity [84]. Together, these findings support a model in which alpha-like subunits constitute an ancient assembly module that has been selectively tuned to meet species- and polymerase-specific regulatory demands, while retaining a conserved dimerization framework essential for polymerase biogenesis.

In plants, additional diversification of alpha-like subunits further illustrates the evolutionary plasticity of this scaffold. *Arabidopsis thaliana* encodes two AC40 paralogs that differentially associate with RNAPI and RNAPIII. These paralogs retain the conserved α-motif heterodimerization interface but exhibit specialized expression patterns, suggesting subfunctionalization following gene duplication [85–87]. The existence of plant-specific alpha-like isoforms demonstrates the adaptability of the ancestral assembly module.

## Disease implications of the RNAPI and RNAPIII alpha-like subunits

**Figure 5.**
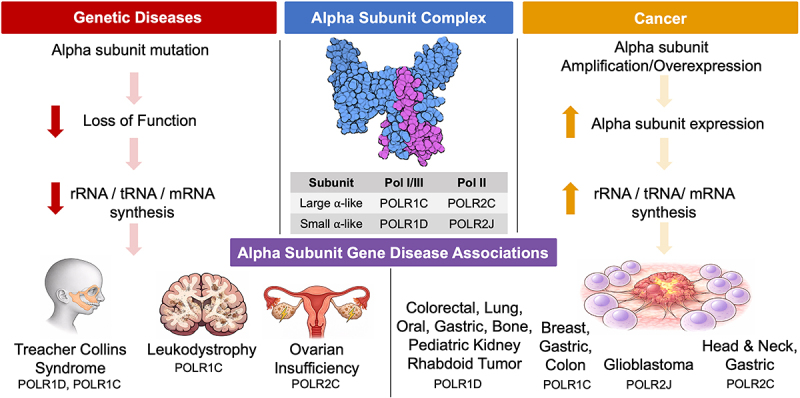
Dosage and disease associations of alpha-like subunits.

Mutations and dysregulation of RNA polymerase subunits are well-established contributors to human disease [7–9] (Figure 5). The alpha-like subunits function at the intersection of RNA polymerase assembly, initiation factor coupling, and subcellular localization. Perturbations in these proteins can disrupt transcription through multiple non-mutually exclusive mechanisms. Diseases linked to alpha-like subunits therefore reflect not only reduced transcriptional output but also defects in RNA polymerase biogenesis, nucleolar organization, and tissue-specific sensitivity to biosynthetic stress. RNAPI and RNAPIII collectively drive ribosome biogenesis and translation capacity and defects in the shared subunits can compromise growth and survival of highly biosynthetic cell types.

Alpha-like subunits are dosage-sensitive assembly scaffolds whose perturbation can disrupt transcription through multiple mechanisms. Loss-of-function mutations that destabilize heterodimerization interfaces impair RNA polymerase assembly or nucleolar localization, reducing RNAPI and/or RNAPIII activity. Such defects underlie ribosomopathies including Treacher Collins syndrome (TCS) and POLR3-related leukodystrophy, which reflect tissue-specific sensitivity to impaired ribosome biogenesis or RNAPIII transcription. Haploinsufficiency of the RNAPII alpha-like subunit POLR2C has been associated with primary ovarian insufficiency, consistent with reduced transcriptional capacity affecting germ cell development. Conversely, increased dosage or amplification of alpha-like subunits, particularly POLR1D and POLR2J, enhances transcriptional output and proliferative signalling. Elevated POLR1D expression promotes rRNA synthesis and activation of β-catenin and Cyclin D1 pathways, supporting oncogenic growth across multiple tumour types.

## Treacher Collins syndrome

Treacher Collins Syndrome (TCS) is a rare craniofacial disease that occurs in approximately 1 in 50,000 live births and is characterized by craniofacial hypoplasia, cleft palate, and ear anomalies [88]. There are four different types of TCS, each affiliated with one of four causative genes. TCS Type 1 (TCS1) is the most common TCS type, caused by loss-of-function mutations in *TCOF1*. *TCOF1* encodes Treacle, a ribosomal biogenesis factor and a key regulator of RNAPI transcription. Additionally, TCS2 is the second most common TCS type, caused by mutations in POLR1D; TCS3 is caused by mutations in *POLR1C*, and the recently described TCS4 is caused by mutations in *POLR1B*. TCS is genetically heterogeneous, with autosomal dominant (TCOF1, *POLR1D*, *POLR1B*) and recessive (*POLR1D*, *POLR1C*) inheritance patterns; dominant mutations also arise *de novo* [89–91]. Many alpha subunit disease-associated variants cluster in the heterodimerization interfaces, indicating that destabilization of the alpha-like scaffold is a common pathogenic mechanism [88].

TCS pathogenesis is strongly linked to impaired ribosome biogenesis, nucleolar stress, and activation of p53-dependent and independent apoptosis, particularly in neural crest-derived progenitors that exhibit high biosynthetic demands during craniofacial development [88,89,92]. Within this framework, POLR1C and POLR1D mutations might reduce RNAPI and RNAPIII activity through impaired assembly, defective initiation factor coupling, or altered localization, ultimately triggering cell death in sensitive developmental lineages.

## Hypomyelinating leukodystrophy

Hypomyelinating leukodystrophy (HLD), also referred to as POLR3-related leukodystrophy or 4H leukodystrophy, is caused by pathogenic variants in multiple RNAPIII subunits and is characterized by hypomyelination, hypodontia, and hypogonadotropic hypogonadism, along with motor and cognitive impairment [93,94]. POLR1C mutations can also cause HLD in addition to their established role in TCS, highlighting the importance of a shared alpha-like subunit in RNAPIII function. Even though the alpha subunits are shared between RNAPI and RNAPIII, there are distinct molecular consequences of alpha-like subunit mutations that can bias disease manifestation towards RNAPIII–dominant or combined RNAPI/III phenotypes. At the molecular level, these mutations often impair RNAP III assembly, stability, or nuclear localization, resulting in reduced transcription and translational capacity [95–97]. Oligodendrocyte lineage cells, which require robust RNAPIII transcription for tRNA and other small RNA production during myelination, may be particularly sensitive to reductions in RNAPIII capacity. These cells must sustain high levels of protein synthesis to support membrane expansion and myelin formation. This heightened demand likely explains the selective vulnerability of white matter in HLD, despite the ubiquitous expression of RNAPIII. More broadly, these findings illustrate how perturbations in shared alpha subunits can give rise to highly cell type–specific disease phenotypes due to differences in cellular demand and functional thresholds.

## Alpha-like subunits and neurodegenerative disease associations

Beyond congenital disorders, altered expression of POLR1C and POLR1D has been reported in several neurodegenerative conditions. For instance, POLR1D expression is downregulated in Parkinson’s disease cohorts [98], while proteomic and genetic association studies have implicated both POLR1D and POLR1C in Alzheimer’s disease risk [99,100]. Although causal relationships remain to be established, these observations align with growing evidence that nucleolar dysfunction and impaired ribosome biogenesis are early cellular features in several neurodegenerative diseases. Given the central roles of RNAPI and RNAPIII in maintaining translational capacity, dysregulation of shared alpha-like subunits may contribute to neuronal and glial vulnerability under conditions of proteostatic or metabolic stress.

## Alpha-like subunits and cancer

Beyond their essential roles in polymerase assembly, alpha-like subunits are increasingly implicated in cancer progression, where elevated expression correlates with poor prognosis across multiple tumour types [80,101–105]. POLR1C is elevated in breast, gastric, and colon cancer [103,106–108] (Figure 5). POLR1D is frequently upregulated in colorectal cancer, and is also upregulated in lung cancer, gastrointestinal cancers, oral squamous cell carcinoma, osteosarcoma, and paediatric rhabdoid tumours of the kidney, consistent with the heightened demand for ribosome biogenesis and noncoding RNA production in rapidly proliferating cancer cells [78,80,109–115] (Figure 5). In colorectal cancer, amplification of the POLR1D gene increases POLR1D dosage, elevating rRNA synthesis and supporting enhanced translational capacity [80,109]. These findings suggest that elevated expression of alpha-like subunits may enhance transcriptional capacity across multiple polymerase systems. Alternatively, their increased expression may reflect the elevated transcriptional demands associated with cancer-associated hypertranscription, a phenomenon recognized in aggressive human malignancies [116,117]. Whether alpha-like subunits actively drive this process or are upregulated as a consequence of increased transcriptional demand remains unclear.

Mechanistic studies further indicate that POLR1D promotes tumour cell proliferation by suppressing p53-mediated cell-cycle arrest through its role in ribosome biogenesis, consistent with alleviation of nucleolar stress surveillance pathways that monitor rRNA synthesis. In this context, reduced rRNA production activates p53-dependent checkpoints, whereas elevated POLR1D expression sustains rRNA synthesis and suppresses this stress response. Beyond this canonical pathway, POLR1D also promotes cell-cycle progression through ribosome biogenesis–independent mechanisms, including increased accumulation of β-catenin and its downstream target Cyclin D1, which drives the G1–S transition [80]. Notably, these effects persist even under conditions where rRNA synthesis is inhibited, suggesting separable roles for POLR1D in proliferative signalling. This dual influence on both ribosome production and cell-cycle regulators suggests that alpha-like subunits can coordinate biosynthetic capacity with proliferative signalling, thereby synchronizing growth and division in cancer cells. Such coupling may explain why modest increases in assembly scaffold abundance can exert disproportionately large effects on tumour progression.

POLR1D has also been linked to the activation of additional growth-promoting pathways, including upregulation of VEGFA and activation of p38 MAPK signalling, as well as upstream regulation by the transcription factor YY1, which enhances POLR1D expression in cancer cells [112,118]. More recently, POLR1D has been shown to localize outside the nucleus and interact with mTORC1 regulatory components, including Rag GTPases, implicating it in nutrient-sensing pathways that coordinate cellular growth with metabolic status [79,109]. However, while these observations support a broader role for POLR1D in proliferative signalling, some of the underlying mechanisms remain incompletely defined. In this context, a separate study demonstrated that, in response to bevacizumab (anti-VEGFA monoclonal antibody) treatment, the chromosomal region 13q12.2 is specifically amplified, leading to increased POLR1D gene dosage. This amplification likely represents a selective adaptation to therapeutic pressure, as increased POLR1D dosage correlates with elevated VEGFA expression. These findings support a model in which POLR1D contributes to bevacizumab resistance by promoting VEGFA expression, thereby functionally counteracting VEGFA neutralization. In turn, increased POLR1D levels may enhance pro-angiogenic signalling beyond the inhibitory capacity of clinically achievable bevacizumab concentrations [118]. Although the precise mechanism by which POLR1D regulates VEGFA expression remains unclear, this relationship further supports a role for POLR1D in coordinating transcriptional and signalling networks that promote tumour adaptation and therapeutic resistance. Such coupling may explain why modest increases in assembly scaffold abundance can exert disproportionately large effects on tumour progression and raises the possibility that POLR1D possesses ‘extra-polymerase’ functions that extend beyond its canonical role in RNAPI and RNAPIII assembly.

## Disease associations of RNAPII alpha-like subunits

Mutations in RNAPII alpha-like subunits are associated with disease. Heterozygous nonsense mutations in RNAPII subunit POLR2C have been associated with primary ovarian insufficiency (POI), a condition involving premature loss of ovarian function [119]. Experimental knockdown of POLR2C reduces cell proliferation and DNA replication, consistent with haploinsufficiency impairing transcriptional programmes required for germ cell development. Disease-associated POLR2C truncations eliminate conserved interfaces required for interaction with the largest RNAPII subunit, suggesting defective polymerase assembly or stability as a contributing mechanism.

Variants in POLR2C have also been reported in association with male infertility and congenital hearing loss, including missense substitutions in conserved structural regions shared across alpha-like subunits. These variants might impair coupling between the alpha-like scaffold and the broader polymerase core, leading to tissue-selective transcriptional insufficiency [120]. POLR2C is also overexpressed in gastric cancer and head and neck squamous cell carcinoma [102,105]. Separately, POLR2J has been reported as upregulated in glioblastoma, where increased expression correlates with poor prognosis and experimental suppression inhibits cell proliferation [104]. These data suggest that, as with RNAPI/III alpha-like subunits, RNAPII alpha-like subunit dosage can influence proliferative capacity and oncogenic potential.

Collectively, disease-associated variants and expression changes in alpha-like subunits underscore the importance of their dual role as both structural assembly factors and regulatory integration points within RNA polymerase complexes. Mutations can impair polymerase biogenesis, alter subcellular localization, or shift transcriptional capacity, with downstream consequences that vary by tissue type and developmental context. This mechanistic versatility provides a unifying explanation for how alterations in highly conserved structural proteins can produce diverse clinical phenotypes ranging from craniofacial malformations and leukodystrophy to infertility and cancer.

## Future perspectives

Despite their central architectural roles, alpha-like subunits remain among the least mechanistically understood components of RNA polymerases. Several key questions now define the next frontier of investigation. First, the molecular choreography of polymerase biogenesis, including when and where heterodimers form, how they engage assembly factors, and how assembly is coordinated with nuclear or nucleolar targeting remains poorly defined, particularly for RNAPI and RNAPIII. Emerging proteomic and imaging approaches capable of tracking assembly intermediates *in vivo* will be essential for resolving these dynamic pathways.

Second, the extent to which alpha-like subunits participate directly in regulatory communication between transcription factors and the polymerase core remains incompletely explored. Structural studies increasingly reveal alpha-like heterodimers positioned at interfaces with initiation factors and polymerase-specific subcomplexes, suggesting that they may function as signal transmission hubs rather than static structural elements. Dissecting how disease-associated variants alter these interfaces, and whether such defects preferentially affect initiation, elongation, or transcriptional responses to stress, will be critical for understanding tissue-selective vulnerability.

From a translational perspective, alpha-like subunits are emerging as potential biomarkers and therapeutic targets. Overexpression of POLR1D and POLR2J in multiple cancers suggests that modest modulation of alpha-like subunit levels or interactions could influence disease progression. However, the feasibility of targeting these scaffolding proteins therapeutically will require deeper understanding of how partial disruption affects polymerase stability versus regulatory function, and whether selective polymerase vulnerabilities can be exploited to treat disease.

## Conclusion

Alpha and alpha-like subunits represent an evolutionarily conserved assembly scaffold that has been repeatedly adapted to support increasingly complex transcription systems. While historically viewed as passive structural components, accumulating evidence demonstrates that these proteins integrate polymerase biogenesis with regulatory interactions, subcellular localization, and transcriptional responsiveness. Their central architectural placement enables small perturbations in stability, interaction surfaces, or expression to propagate broadly through transcriptional networks. By unifying structural, biochemical, evolutionary, and clinical perspectives, this review highlights alpha-like subunits as critical determinants of RNA polymerase function and emerging contributors to transcription-linked pathologies. Continued investigation of these conserved scaffolds promises not only to refine models of polymerase assembly and regulation but also to uncover new principles governing how transcriptional machinery adapts to cellular stress and developmental demand.

## Data Availability

No new data were created or analysed in this study. Data sharing is not applicable to this review article.
